# Comparison of pre-operative Hirschsprung-associated enterocolitis using classical criteria and Delphi method: A diagnostic study

**DOI:** 10.1016/j.amsu.2020.01.007

**Published:** 2020-02-03

**Authors:** Gunadi, Hapsari Hayu Ningtyas, Susan Simanjaya, Maharani Febrianti, Fiko Ryantono, Akhmad Makhmudi

**Affiliations:** Pediatric Surgery Division, Department of Surgery, Faculty of Medicine, Universitas Gadjah Mada/Dr. Sardjito Hospital, Yogyakarta, 55281, Indonesia

**Keywords:** Hirschsprung disease, Pre-operative HAEC, Delphi method, Classical criteria

## Abstract

**Background:**

Hirschsprung-associated enterocolitis (HAEC) is the most common complication of Hirschsprung disease (HSCR) that may happen pre-operatively. Several methods have been reported to determine HAEC. Because the diagnosis of pre-operative HAEC might change the surgical plan, we aimed to determine the accuracy of the classical criteria for diagnosis of pre-operative HAEC and using the Delphi method as a gold standard.

**Methods:**

Medical records of HSCR children who were admitted to our hospital from January 2009 to December 2015 were retrospectively analyzed.

**Results:**

Ninety-six subjects were involved in this study, consisting of 74 males and 22 females. The most common findings of the Delphi score were abdominal distension (100%) and dilated loops of bowel (100%), followed by leucocytosis (78.6%), lethargy (71.4%), cutoff sign in rectosigmoid with absence of distal air (71.4%), and shift to left (71.4%). The frequency of pre-operative HAEC was 4.2% and 14.6% using the classical criteria and Delphi method, respectively (*p* = 0.016). The sensitivity, specificity, positive predictive value, negative predictive value, and accuracy rates of the classical criteria for diagnosis of pre-operative HAEC were 14.3% (95% CI: 1.8–42.8%), 97.6% (95% CI: 91.5–99.7%), 50% (95% CI: 13.3–86.7%), 87% (95% CI: 84.3–89.2), and 85.4% (95% CI: 76.7–91.8%), respectively.

**Conclusions:**

The frequency of pre-operative HAEC is low in our hospital. The accuracy of the classical criteria is considered relatively moderate for diagnosis of pre-operative HAEC.

## Highlights

• Diagnosis of pre-operative Hirschsprung-associated enteroclitis (HAEC) might change surgical plan for Hirschsprung disease.
• The accuracy of the classical criteria is considered relatively moderate for diagnosis of pre-operative HAEC.
• It is necessary to develop the HAEC diagnosis guidelines to make it more standardized, clinically relevant, and easy to use.

## 1 Introduction

Hirschsprung disease (HSCR) is a complex genetic disorder that is caused by the absence of intramural ganglion cells in the Auerbach and Meissner plexus, resulting in a functional obstruction of the colon [1]. Hirschsprung-associated enterocolitis (HAEC) is one of the most dangerous and life-threatening complications of HSCR. HAEC can be avoided by diagnosing HSCR timely and accurately [2]. The classical diagnostic criteria were formerly used to diagnose HAEC [3], which are based on the clinical presentations that are most often seen in the clinical setting, such as abdominal distension (83%), explosive diarrhea (69%), vomiting (51%), fever (34%), lethargy (27%) rectal bleeding (5%) and colonic perforation (2.5%) [3]. Radiological examination revealing a cut-off appearance is also included as one of the criterion in the classical diagnostic criteria. Cutoff appearance in radiological examination is sensitive and specific for HAEC. Thus, a child is diagnosed with HAEC when there is abdominal distension, explosive diarrhea and cut-off appearance in the radiological analysis [3].

The incidence of HAEC has been reported to be in the range of 6–26% before the definitive operation or during the diagnosis of HSCR and ranges 5–42% post-operatively [4]. Besides that, there were also reports of HAEC occurring as often as in 15.5–20.8% of their cases [5,6]. This variation is presumably the result of the lack of standardized guidelines for diagnosing HAEC. HAEC had previously been diagnosed solely based on non-specific clinical criteria. The Delphi method was developed to diagnose HAEC by identifying the relevant clinical diagnostic criteria of HAEC [7].

Diagnosing HAEC prior to surgery may have an impact on the surgical planning of the child. Once the pre-operative HAEC established, there will be a treatment toward alleviating its acute symptoms, including hydration, metronidazole, broad spectrum antibiotic, rectal irrigations or colostomy. These treatments depend on its severity [8]. With the change of diagnosis possibly affecting the surgical plan, we aimed to determine the accuracy of the classical criteria for diagnosis of pre-operative HAEC and using the Delphi method as a gold standard.

## 2 Methods

### 2.1 Subjects

This research was a retrospective study using medical records of HSCR children admitted to our hospital from January 2009 until December 2015. We collected 140 medical records of HSCR children and excluded 44 due to incomplete data. A total of 96 children were analyzed in this study (Table 1).

**Table 1:** Characteristics of HSCR children admitted to our hospital.

Characteristics	Classical criteria		Delphi method		Total (n = 96)
	HAEC	Non-HAEC	HAEC	Non-HAEC	
Sex					
⁃ Male	3	71	11	63	74
⁃ Female	1	21	3	19	22
Age (months)					
⁃ Median (IQR)	27.6 (1.5–39.6)	2.8 (0.6–11.1)	7.7 (1.0–74.4)	2 (0.6–12.0)	2.8 (0.6–13.5)
Aganglionosis type					
⁃ Short	4	86	13	77	90
⁃ Long	0	6	1	5	6

HAEC, Hirschsprung-associated enterocolitis; IQR, interquartile range.

This study was approved by the Ethical Committee of our institution (Ref. KE/FK/787/EC/2015). Informed written consent has been signed by patients’ parents.

### 2.2 Classical criteria and Delphi method

The data collection was done by using checklists containing the classical criteria and Delphi method criteria along with its scoring. The diagnosis of HAEC was determined using both the classical criteria and the Delphi methods. The Delphi method is consisting of: a) history (diarrhea with explosive stool, diarrhea with foul-smelling stool, diarrhea with bloody stool, and history of enterocolitis); b) physical examination (explosive discharge of gas and stool on rectal examination, distended abdomen, decreased peripheral perfusion, lethargy, and fever); c) radiologic examination (multiple air fluid levels, dilated loops of bowel, sawtooth appearance with irregular mucosal lining, cutoff sign in rectosigmoid with absence of distal air, and pneumatosis); and d) laboratory findings (leucocytosis and shift to left) (Table 2). Children are diagnosed with HAEC when they have a score of ten or greater [7,9,10].

**Table 2:** Frequency of each criterion of HAEC children admitted to our hospital based on the Delphi Method.

Criteria	Frequency of pre-operative HAEC
History	
• Diarrhea with explosive stool	5/14 (37.4%)
• Diarrhea with foul-smelling stool	5/14 (37.4%)
• Diarrhea with bloody stool	1/14 (70.1%)
• History of enterocolitis	7/14 (50.0%)
Physical examination	
• Explosive discharge of gas and stool on rectal examination	8/14 (57.1%)
• Distended abdomen	14/14 (100%)
• Decreased peripheral perfusion	4/14 28.6%)
• Lethargy	10/14 71.4%)
• Fever	8/1457.1%)
Radiologic Examination	
• Multiple air fluid levels	9/14 (63.4%)
• Dilated loops of bowel	14/14 (100.0%)
• Sawtooth appearance with irregular mucosal lining	2/14 (14.3%)
• Cutoff sign in rectosigmoid with absence of distal air	10/14 (71.4%)
• Pneumatosis	1/14 (7.1%)
Laboratory	
• Leucocytosis	11/14 (78.6%)
• Shift to left	10/14 (71.4%)

When using the classical diagnostic criteria, a child is determined to have HAEC when fulfills the following criteria: abdominal distension, explosive diarrhea and cutoff appearance upon radiological examination [3].

### 2.3 Statistical analysis

The data obtained from this study were presented as median (interquartile range [IQR]) and percentages that reflect the frequency of HAEC as diagnosed by the two means of clinical diagnosis. The classical criteria test was analyzed for sensitivity, specificity, positive predictive value, negative predictive value, and accuracy, while the Cohen's Kappa concordance coefficient and McNemar test were applied to determine the sensitivity and specificity differences between the measurements [11]. All statistical analysis was done using the IBM Statistical Package for Social Science (SPSS) version 21 (IBM Corp., Chicago).

## 3 Results

Among 96 HSCR children, there was a male preponderance (77.1%), with the median age of the subjects of 2.8 (IQR, 0.6–13.5) months. Additionally, only six out of the ninety-six (6.3%) subjects had long type aganglionosis (Table 1).

The most common findings of the Delphi score were abdominal distension (100%) and dilated loops of bowel (100%), followed by leucocytosis (78.6%), lethargy (71.4%), cutoff sign in rectosigmoid with absence of distal air (71.4%), and shift to left (71.4%) (Table 2).

Based on the Delphi method, 14/96 (14.6%) of the children had HAEC before the surgery with a mean score of 10.79 ± 1.85. Among those diagnosed as HAEC with the Delphi method, eleven were male and three were female while only one of them had long type aganglionosis (Table 1).

Meanwhile, based on the classical criteria, only 4/96 (4.2%) children were diagnosed with HAEC before the surgery. All four of the HAEC children had short type aganglionosis. Among these four children, there were three males and one female (Table 1).

The sensitivity, specificity, positive predictive value, negative predictive value, and accuracy rates of the classical criteria for diagnosis of pre-operative HAEC were 14.3% (95% CI: 1.8–42.8%), 97.6% (95% CI: 91.5–99.7%), 50% (95% CI: 13.3–86.7%), 87% (95% CI: 84.3–89.2), and 85.4% (95% CI: 76.7–91.8%), respectively (Table 3).

**Table 3:** Comparison of frequency of pre-operative HAEC between the classical criteria and Delphi method in HSCR children at our hospital.

		Delphi Method	
		HAEC (+)	HAEC (−)
**Classical Criteria**	HAEC (+)	2	2
	HAEC (−)	12	80

HAEC, Hirschsprung-associated enterocolitis; HSCR, Hirschsprung disease.

McNemar test showed that the sensitivity and specificity rates was significantly different between classical criteria and Delphi method (*p* = 0.016), whereas the Cohen's Kappa index was 0.17 (slight agreement).

## 4 Discussion

This study was conducted to evaluate the classical diagnostic criteria for diagnosis of pre-operative HAEC. We showed that the classical criteria had a relatively moderate accuracy as a diagnostic tool for pre-operative HAEC.

Moreover, our findings revealed that the frequency of pre-operative HAEC was significantly different between two tests. This may be attributed to the very restricted criteria that are contained within the classic diagnostic criteria which do not take into account other clinical signs and symptoms such as fever, vomiting, et cetera which are also relevant for HAEC. Besides that, despite already including radiological data, the criteria still lack the utilization of laboratory results. Thus, this difference makes the classical diagnostic criteria less sensitive (~14%) for diagnosing pre-operative HAEC. The Delphi method uses criteria that were specifically developed and designed to diagnose HAEC [7]. Several studies used Delphi method to evaluate HAEC in their patients [5,9,10,12]. However, the criteria are long and complicated to be adopted in the daily practice such that it has not yet been adopted and applied universally [8]. It has been proposed to utilize a cutoff of 4 rather than 10 to increase Delphi method's sensitivity and specificity [5,12]. In addition, recently the American Pediatric Surgical Association Board of Governors has established the guidelines for diagnosis of HAEC to make it more rational, standardized, clinically relevant, and easy to use [9].

Previously, there have not been any studies that compare the Delphi method and classical diagnostic criteria in diagnosing HAEC before definitive surgery. Moreover, the frequency of HAEC yielded in this study (14.6%) using the Delphi method is lower than that reported by Frykman et al. (37.1%) [5] and Dore et al. (22.6%) [12]. These discrepancies are a result of the different cutoff of Delphi method used. Frykman et al. [5] and Dore et al. [12] diagnosed HAEC by using the cutoff of 4, while our study determined the diagnosis of HAEC by using the cutoff of 10. Furthermore, Gosain et al. described that the clinical manifestation that was used to diagnose HAEC was not specific such that it could suggest other diseases that eventually would delay the diagnosis of HAEC [13]. Interestingly, when lowering the cutoff of Delphi method to 2, its sensitivity to detect HAEC even higher that it would catch children with slight clinical manifestations of HAEC [5].

In this study, we compared the accuracy of classical method for diagnosis of pre-operative HAEC and using the Delphi method as a gold standard. Unfortunately, we were unable to determine the period of follow up and the effect of using any of the classic or Delphi methods on HSCR patient's outcome and quality of life because our report was a retrospective study that extracting data from medical records, becoming a limitation of our study.

Another weakness of this report is that the radiological data of many children (~30%) admitted from the year 2009 till 2013 were not complete, therefore, the diagnosis of pre-operative HAEC could have been affected and the incidence rate appeared to be lower. Notably, this report is a single center retrospective study. This fact should be considered during the analysis of our findings.

In conclusions, the frequency of pre-operative HAEC is low in our hospital. The accuracy of the classical criteria is considered relatively moderate for diagnosis of pre-operative HAEC.

## Ethical approval

This study has been approved by the Ethical Committee of Faculty of Medicine, Public Health and Nursing, Universitas Gadjah Mada/Dr. Sardjito Hospital (Ref: KE/FK/787/EC/2015).

## Sources of funding

A grant was given by the Indonesia Ministry of Research, Technology and Higher Education (#2817/UN1.DITLIT/DIT-LIT/LT/2019 to G.).

## Author contribution

Gunadi, Hapsari Hayu Ningtyas, and Akhmad Makhmudi brainstormed this study. Hapsari Hayu Ningtyas, Susan Simanjaya, Maharani Febrianti and Fiko Ryantono prepared the manuscript draft and Gunadi critically revised the manuscript for important intellectual content. Gunadi, Hapsari Hayu Ningtyas, Susan Simanjaya, Maharani Febrianti, Fiko Ryantono and Akhmad Makhmudi facilitated all project-related tasks. All authors read and approved the final manuscript.

## Registration of research studies

1. Name of the registry: Research Registry
2. Unique Identifying number or registration ID: researchregistry5218
3. Hyperlink to the registration (must be publicly accessible): https://www.researchregistry.com/browse-the-registry#home/registrationdetails/5dc50e541e687b001587986a/

## Guarantor

Gunadi.

## Consent

Informed written consent has been signed by patients’ parents.

## Patient consent

Informed written consent has been signed by patients’ parents.

## Provenance and peer review

Not commissioned, externally peer reviewed.

## Declaration of competing interest

No potential conflict of interest relevant to this article was reported.
